# Complete coding sequence of Taiwan bat lyssavirus 2 from Chinese noctule (*Nyctalus plancyi velutinus*) at New Taipei City, Taiwan

**DOI:** 10.1128/mra.00723-26

**Published:** 2026-09-16

**Authors:** Shu-Chia Hu, Chao-Lung Hsu, Chao-Chin Chang, Fan Lee

**Affiliations:** 1 Veterinary Research Institute, Ministry of Agriculture 71682, New Taipei City, Taiwan; 2 Bat Conservation Society of Taipei, Taipei City, Taiwan; Portland State University, Portland, Oregon, USA

**Keywords:** bat, *Lyssavirus*, Taiwan, *Nyctalus plancyi*

## Abstract

The complete coding sequence of Taiwan bat lyssavirus 2 was obtained from Chinese noctule (*Nyctalus plancyi velutinus*) at Zhonghe District, New Taipei City, Taiwan, in 2024. Sequence comparison and phylogenetic analysis revealed that the genome was closely related to those lyssaviruses within Phylogroup I.

## ANNOUNCEMENT

The genus *Lyssavirus* of the family *Rhabdoviridae* is composed of 19 species (1). One of the members in the genus, Taiwan bat lyssavirus 1 (*Lyssavirus formosa*, TWBLV-1), was isolated from Japanese pipistrelles (*Pipistrellus abramus*) in 2016 and 2017 (2). Another candidate of the genus, Taiwan bat lyssavirus 2 (TWBLV-2), was later isolated in 2018 from a *Nyctalus plancyi velutinus* (3), an insectivorous bat distributed in mainland China and Taiwan (4).

The complete coding sequence reported in this study was a virus identified from the brain tissue of an *N. p. velutinus*, which was found dead at Zhonghe District, New Taipei City, Taiwan, in 2024. The bat was submitted for surveillance on lyssaviruses and was tested positive by direct fluorescent antibody test and RT-PCR, as previously described (2). Its brain tissue was homogenized into 10% (wt/vol) brain homogenate in minimum essential medium (Gibco, Thermo Fisher Scientific, USA), and the supernatant was used for nucleic acid extraction after centrifugation at 1,016 × *g* for 10 min. The supernatant was extracted by magLEAD 12gC Automated Nucleic Acid Extraction System (Precision System Science Co., Ltd., Japan). The genomic sequence of the virus isolate (designated as TWBLV-2/NT/2024) was obtained as previously described (3). Virus sequences were amplified by RT-PCR with the 12 primer sets (Table 1) and the kit SuperScript III One-Step RT-PCR System with Platinum Taq High Fidelity (Invitrogen, Thermo Fisher Scientific). The RT-PCR product was sequenced with the 3730xl DNA Analyzer (Applied Biosystems, Life Technologies, USA) by a commercial service (Mission Biotech, Taiwan). The consensus sequence for each RT-PCR amplicon was generated from assembled forward and reverse reads using SeqMan Ultra (version 19.0.1; DNASTAR Lasergene, USA). Poor-quality or ambiguous amplicons were cloned into the pCRII-TOPO vector (Invitrogen) and Sanger sequenced to obtain reliable consensus sequences. The genomic sequence was then assembled from overlapping the consensus sequences using SeqMan Ultra. Sequence identity analyses were performed using BioEdit software (version 7.0.5.3) (5).

**TABLE 1:** Primers used in the whole genome sequencing in this study

Primer (reference no.)	Primer sequence, 3′–5′
TWBLV 1F (2)	ACGCTTAACGACAAAA YC
TWBLV 1R (2)	TCTTGCATTTCTTTCTCATC
TWBLV-2-2F (3)	TTTGTGGGTTGCTATATGGG
TWBLV-2-2R (3)	GAGAAATATCCCGGAGGACC
TWBLV 3F (2)	AGARATAGCWCATCAGATWGC
TWBLV 3R (2)	CTATTGTGTGGCACCAT WAC
TWBLV-2-4F (3)	GATGAGGACAAGAACACCTC
TWBLV-2-4R (3)	TTCTAAAATGACTGAGCCTCC
TWBLV 5F (2)	CTGATGGAYGGRTCATGGGT
TWBLV-2-5R (3)	GAAAGGTGAACAGTTGAAAT
TWBLV 6F (2)	AACAGGTAGCTCCCGAGTTTGTTC
TWBLV-2-6R (3)	CTGAATGAGATCCGTGTATCCACA
TWBLV 7F (2)	ACTGAGGTTTATGATGACCC
TWBLV 7R (2)	CCCCAGTGTCTATA RCAWCC
TWBLV-2-8F (3)	CATTCTTTGGGTGACTTTCC
TWBLV-2-8R (3)	CATATAAGTCGGGCATAAG
TWBLV 9F (2)	CATGCTGGAACGGTCAGGA YG
TWBLV-2-9R (3)	CCGAATTAAAAAAGGACTCTT
TWBLV 10F (2)	CTCAGTGAGTT RTTYAGCTC
TWBLV-2-10R (3)	CAAATGGATGAACCTATT
TWBLV 11F (2)	CATGATTCAGGGTA YAAYGA
TWBLV 11R (2)	GTCTGTAACTTCTGCATCAC
TWBLV 12F (2)	ATCTGGGAAAAGCCATCAGA
TWBLV 12R (2)	ACGCTTAACAAAAAAAACAA

**Fig 1:**
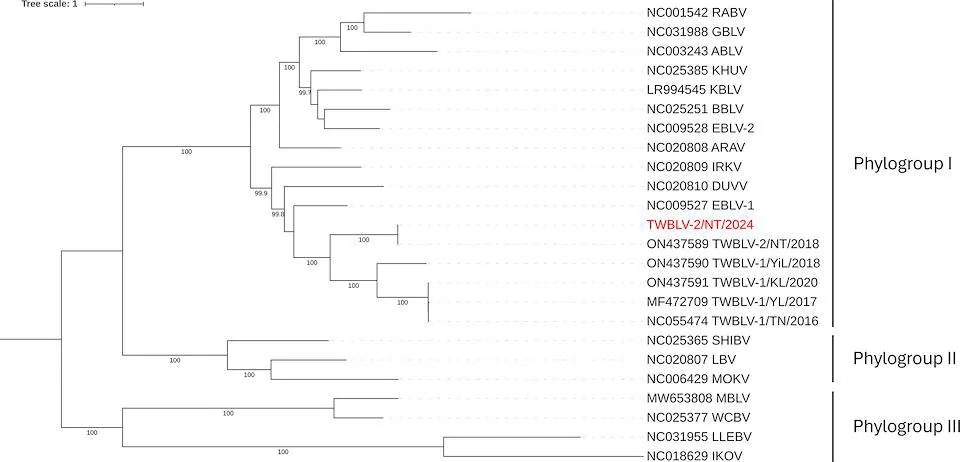
Phylogenetic relationship of TWBLV-2/NT/2024, marked in red letters, identified in a *Nyctalus plancyi velutinus* in 2024, Taiwan. The concatenated coding sequences of representatives of all *Lyssavirus* species from GenBank were analyzed, and their GenBank accession numbers were provided in front of the viruses’ abbreviations. The sequences were aligned using the MUSCLE program implemented in the software Molecular Evolutionary Genetic Analysis (version X) (6). The maximum likelihood phylogeny was constructed with concatenated coding sequences by IQ-TREE (7) using the best-fit model of nucleotide substitution with 1,000 ultrafast bootstrap (8). Numbers (over 95%) at the nodes indicate ultrafast bootstrap supports. The lyssaviruses of Phylogroup III were used as the outgroup. The phylogenetic tree produced by IQ-TREE was further edited and visualized using the Interactive Tree of Life (iTOL) online platform (https://itol.embl.de/) (9).

The genome of the TWBLV-2/NT/2024 was 11,917 bases long, with 43.26% GC content. The concatenated coding sequences of the TWBLV-2 isolates obtained in 2018 (3) and in the present study showed a high nucleotide identity (99.7%) with each other and shared only 75.4%–79.8% nucleotide identities with TWBLV-1 isolates. Our phylogenetic analysis indicated that the two TWBLV-2 isolates formed a distinct lineage, separated from other lyssaviruses, and belonged to Phylogroup I (Fig. 1). According to the species criteria of *Lyssavirus* by the ICTV, TWBLV-2 is believed to be a novel *Lyssavirus*, and *N. p. velutinus* may be its reservoir host.

## Data Availability

The raw Sanger sequencing chromatogram files (.ab1) and corresponding amplicon consensus sequences have been deposited in Figshare and are publicly available at https://doi.org/10.6084/m9.figshare.33261528. The GenBank accession number for the assembled genomic sequence is PZ319659.
